# The CD200 Regulates Inflammation in Mice Independently of TNF-α Production

**DOI:** 10.3390/ijms22105358

**Published:** 2021-05-19

**Authors:** Katarzyna Tonecka, Agata Braniewska, Zofia Pilch, Zuzanna Sas, Marcin Skorzynski, Elisabetta Manuali, Tomasz P. Rygiel

**Affiliations:** 1 Department of Immunology, Medical University of Warsaw, 02-097 Warsaw, Poland; k.roszczenko@gmail.com (K.T.); agata.braniewska@gmail.com (A.B.); zofia.pilch@gmail.com (Z.P.); sas.zuza@gmail.com (Z.S.); mskorzynski@wum.edu.pl (M.S.); 2 Laboratory of Veterinary and Comparative Histopathology, Istituto Zooprofilattico Sperimentale Umbria e Marche “Togo Rosati”, 06126 Perugia, Italy; e.manuali@izsum.it

**Keywords:** inflammatory bowel disease, inflammation, immune regulation, myeloid cells, CD200, CD200R

## Abstract

Inflammatory bowel disease is characterized by the infiltration of immune cells and chronic inflammation. The immune inhibitory receptor, CD200R, is involved in the downregulation of the activation of immune cells to prevent excessive inflammation. We aimed to define the role of CD200R ligand-CD200 in the experimental model of intestinal inflammation in conventionally-reared mice. Mice were given a dextran sodium sulfate solution in drinking water. Bodyweight loss was monitored daily and the disease activity index was calculated, and a histological evaluation of the colon was performed. TNF-α production was measured in the culture of small fragments of the distal colon or bone marrow-derived macrophages (BMDMs) cocultured with CD200^+^ cells. We found that *Cd200*^−/−^ mice displayed diminished severity of colitis when compared to WT mice. Inflammation significantly diminished CD200 expression in WT mice, particularly on vascular endothelial cells and immune cells. The co-culture of BMDMs with CD200^+^ cells inhibited TNF-α secretion. In vivo, acute colitis induced by DSS significantly increased TNF-α secretion in colon tissue in comparison to untreated controls. However, *Cd200^−/−^* mice secreted a similar level of TNF-α to WT mice in vivo. CD200 regulates the severity of DSS-induced colitis in conventionally-reared mice. The presence of CD200^+^ cells decreases TNF-α production by macrophages in vitro. However, during DDS-induced intestinal inflammation secretion of TNF-α is independent of CD200 expression.

## 1. Introduction

Inflammatory bowel disease (IBD) is a chronic autoimmune disorder of the gastrointestinal tract that affects the small intestine and/or the colon comprising two main forms, Crohn’s disease (CD) and ulcerative colitis (UC). It is generally accepted that IBD results from an abnormal and extensive immune response to the intestinal flora, however, the etiology of this inflammatory disorder remains unclear. Genetic susceptibility as well as environmental and microbial factors are involved in the pathogenesis of IBD [1].

TNF-α is a very important cytokine that orchestrates the pathogenesis of IBD [2]. It stimulates the production of IL-1β, IL-6, and IL-33, furthermore it regulates the expression of adhesion molecules, fibroblast proliferation, and initiation of cytotoxic, apoptotic, and acute-phase responses [3]. Serum levels of TNF-α correlate with the clinical symptoms in UC and CD patients [4,5]. The importance of TNF-α in colonic inflammation is emphasized by the efficacy of TNF-α blocking therapies in IBD. Despite this therapeutic success in the part of the patients, the rate of surgical interventions has not decreased, pointing out the need for new therapies. One of the main sources of TNF-α in IBD are innate immune cells, such as monocytes and macrophages [6]. In IBD patients, as well as in experimental animal models of intestinal inflammation, circulating blood monocytes are recruited to the intestinal mucosa and differentiate locally into diverse subsets of macrophages with inflammatory properties [7,8,9,10,11,12,13,14]. The experimental model of acute colitis initiated by administration of dextran sulfate sodium (DSS), it is a well established and displays many features of human inflammatory bowel diseases (IBD). DSS leads to enterocyte death and disruption of the colon epithelial barrier, which results in tissue exposure to luminal bacteria and the development of rapid innate immune response. In this model of colitis, bacteria and/or bacterial products are essential for the initiation of an inflammatory response. Germ-free mice exhibit only minor signs of mucosal inflammation upon DSS administration [15,16]. On the contrary, Kitajima et al. has shown that lack of intestinal flora results in increased episodes of death, when mice are administered with 5% DSS in comparison to conventional mice, suggesting that intestinal flora may also play a protective role in DSS-colitis [17]. Recently, it was shown that the bacterial product Pam3CSK4, a TLR2/1 agonist, protects from DSS-induced colitis by manipulating the activity of intestinal macrophages [18].

In a healthy intestine, macrophages play an essential role in maintaining steady-state tissue homeostasis, by the clearance of apoptotic cells and production of growth factors that are essential for epithelial renewal and integrity, as well as their ability to engulf and kill commensal bacteria penetrating the epithelium [19]. The activity of macrophages and T cells can be manipulated, using e.g., immune checkpoint receptors, like CD200R. The inhibitory receptor CD200R is predominantly expressed in cells from the myeloid lineage, such as macrophages, dendritic cells, neutrophils, monocytes, or mast cells, whereas its canonical ligand (CD200) is expressed by a variety of cells, including lymphoid cells, vascular endothelial cells, neurons, kidney glomeruli, tonsil follicles and smooth muscle [20,21,22,23,24,25,26]. Another possible intestinal ligand for CD200R is iSEC1 which may negatively regulate mucosal immune responses [27]. CD200R has been implicated in the downregulation of myeloid cell function via inhibition of pro-inflammatory mediators expression, like TNF-α, IFNs, IL-6, and iNOS, in effector cells [21,22,28,29]. Thus, the CD200-CD200R axis is thought to prevent an excessive immune response in microbial infections. A significant decrease in the number of CD200R expressing immune cells was observed in IBD patients [30,31], suggesting that disruption of CD200-CD200R axis may be involved in the pathogenesis of intestinal inflammation. Previously, we showed that absence of CD200-CD200R interaction improves the clearance of mouse hepatitis coronavirus (MHV) via TLR7 dependent type I interferon (IFN) production [32]. Recently, we showed that TLR7 stimulation changes the tumor microenvironment to become pro-inflammatory and less supportive for tumor growth while decreasing CD200R expression on myeloid cells [33]. Here, we studied the CD200-CD200R axis in DSS-mediated inflammation in mice.

## 2. Results

### 2.1. Lack of CD200 Diminishes the Severity of DSS-Induced Colitis

**Figure 1:**
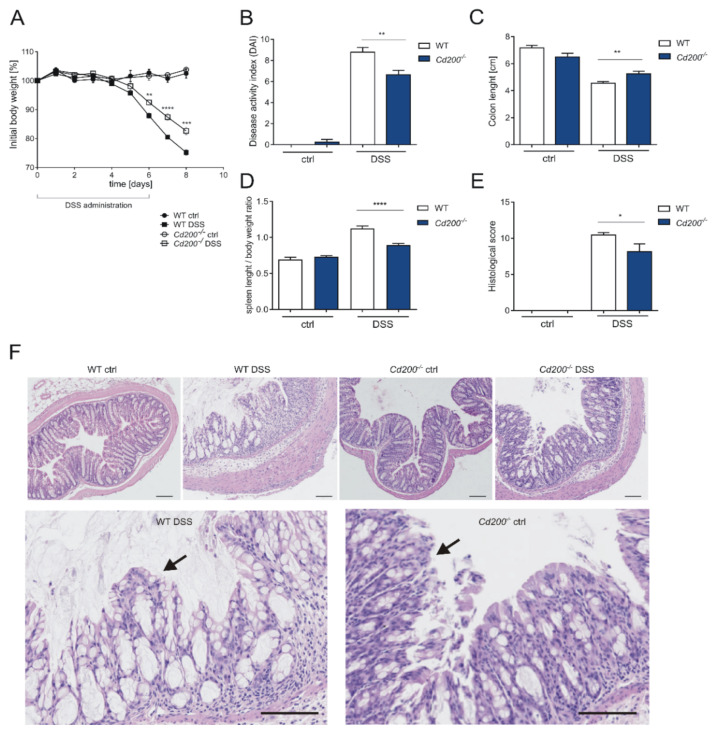
Characteristic of DSS-mediated intestinal inflammation in WT and *Cd200^−/−^* conventionally reared mice. (**A**) Body weight, expressed as the percentage of the initial body weight, n = 4–16; (**B**) Disease activity index (DAI) assessed on day 8, based on the changes in the following parameters: body weight loss, colon length, stool consistency, stool blood, and presence of rectal bleeding, n = 4–14; Length of the colon (**C**) and spleen (**D**) from control or DSS-treated WT and *Cd200^−/−^* mice. C. n = 4–23 D. n = 4–16; (**E**) Histological score of mucosal damage assigned to the different groups, n = 4–9; (**F**) Representative histological sections of colonic mucosa isolated from control or DSS-treated WT and *Cd200^−/−^* mice after HE staining. Scale bar = 100 µm. Results are expressed as mean ± SEM. Significant differences were calculated using Student’s *t*-test * *p* < 0.05. ** *p* < 0.01. *** *p* < 0.001 **** *p* < 0.0001 compared with WT controls. Data shown are combined from two independent experiments.

To evaluate the role of the CD200-CD200R pathway in intestinal inflammation, wild type (WT) and *Cd200^−/−^* mice were administered 3% DSS in drinking water for 6 days. After 4 days of DSS administration, mice exhibited signs of intestinal inflammation, defined as diarrhea and body weight loss. At day 6, WT mice had lost more relative body weight (87.8 ± 0.8% of initial body weight) than *Cd200^−/−^* mice (92.4 ± 0.8% of initial body weight) (*p* < 0.01, *t*-test). On the last day of the experiment (day 8), WT mice had 75.2 ± 1.0% and *Cd200^−/−^* mice had 82.6 ± 1.2% of initial body weight (*p* < 0.001, *t*-test) (Figure 1A). The disease activity index (DAI), which is based on the main features of colitis: weight loss, colon shortening, stool consistency, stool blood, and rectal bleeding, was decreased in *Cd200^−/−^* mice in comparison to WT mice (6.6 ± 0.4 vs. 8.7 ± 0.4, (*p* < 0.01, *t*-test) (Figure 1B). Moreover, the aggravation of the disease outcome was associated with the shorter colon in WT mice (4.5 ± 0.1 cm) in comparison to *Cd200^−/−^* mice (5.2 ± 0.1 cm) (*p* < 0.01, *t*-test) (Figure 1C). Similarly, the increase in average spleen size was smaller in *Cd200^−/−^* mice (14.3 ± 0.2 mm) than in WT mice (16.3 ± 0.4 mm) (*p* < 0.001, *t*-test) (Figure 1D). Concomitantly, DSS-induced colitis caused superficial inflammation of the colon with mucosal damage in DSS-treated WT and *Cd200^−/−^* mice. Histological changes and the distribution of lesions were more severe in WT mice (10.4 ± 0.3) as compared to *Cd200^−/−^* mice (8.1 ± 1.1) (*p* < 0.05, *t*-test) (Figure 1E). Upon induction of colitis, WT mice showed a mild infiltration of inflammatory cells into the mucosa, submucosa and muscular/serosa layers, whereas in *Cd200^−/−^* mice only the mucosal surface was infiltrated. Also, the epithelial damage was more pronounced in the WT group. The histological changes were exemplified by the mucosal erosion with small-sized ulcers, decreased number of goblet cells, and complete crypt loss (represented as total histological score) (Figure 1F, panels WT DSS and *Cd200^−/−^* DSS). No epithelial alterations nor mucosal inflammation were detected in control colon samples of untreated WT and *Cd200^−/−^* mice. Overall, these results suggest, that in conventionally reared mice lack of CD200 decreased susceptibility to chemically-induced colitis.

### 2.2. DSS Increases the Infiltration of Myeloid Cells in the Colon That Is Further Increased in Cd200^−/−^ Mice

**Figure 2:**
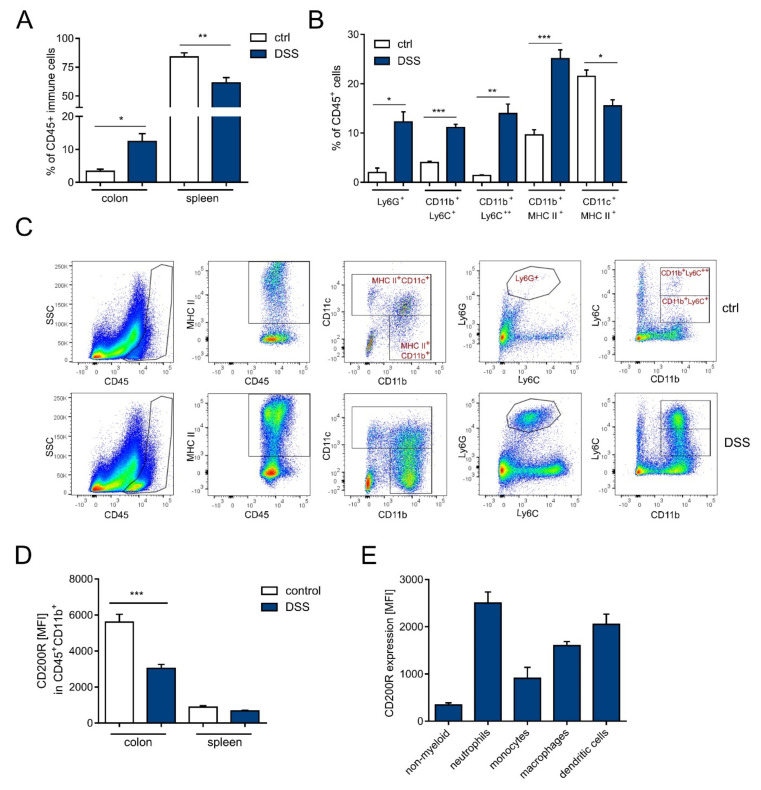
Analysis of immune cell infiltration in colon and spleen during DSS-induced inflammation. (**A**) Frequency of immune cells (CD45^+^) in colon and spleen in DSS-treated and control mice, n = 4–7; (**B**) Percentage of neutrophils (Ly6G^+^), monocytes (CD11b^+^Ly6C^+^ and CD11b^+^Ly6C^++^), macrophages (CD11b^+^CD11c^−^MHC-II^+^), and dendritic cells (CD11b^−^ CD11c^+^MHC-II^+^) in CD45^+^ immune cells in colons of DSS-treated and control mice, n = 3–8; (**C**) Representative dot plots of myeloid cell populations in colons isolated from DSS-treated and control mice; (**D**) Expression of CD200R in CD45^+^CD11b^+^ cells isolated from colons and spleens of DSS-treated or control mice, n = 4–7; (**E**) Expression of CD200R in colonic populations of immune cells, n = 6. All results are expressed as mean ± SEM. Significant differences were calculated using Student’s *t*-test * *p* < 0.05. ** *p* < 0.01. *** *p* <0.001 compared with controls. Data shown are combined from two independent experiments. MFI: mean fluorescence intensity.

DSS-induced colitis is typically associated with an infiltration of immune cells into the large intestine [34]. Indeed we observed an increased frequency of immune cells in the inflamed colon (12.3 ± 2.3%) when compared to untreated controls (3.3 ± 0.6%) (*p* < 0.05, *t*-test) (Figure 2A). Conversely, the percentage of immune cells in spleen was decreased in response to DSS-treatment (61.31 ± 4.67% as compared to untreated controls 83.8 ± 3.6% (*p* < 0.05, *t*-test) (Figure 2A). The immune infiltrate in inflamed intestine consisted of CD11b^+^ myeloid cells, namely neutrophils (CD45^+^Ly6G^+^) 12.2 ± 2.0% vs. control 1.9 ± 0.9% (*p* < 0.05, *t*-test), monocytes (CD11b^+^Ly6C^+^) 11.1 ± 0.6% vs. control 4.0 ± 0.2% (*p* < 0.001, *t*-test) monocytes (CD11b^+^Ly6C^++^) 13.9 ± 1.9% vs. control 1.3 ± 0.1% (*p* < 0.01, *t*-test) and macrophages (CD11c^-^CD11b^+^MHC-II^+^) 25.0 ± 1.7% vs. control 9.6 ± 1.0% (*p* < 0.001, *t*-test). Conversely, the percentage of dendritic cells (CD11c^+^MHC-II^+^) was decreased 15.5 ± 1.2% vs. control 21.5 ± 1.2% (*p* < 0.05, *t*-test) (Figure 2B,C). To evaluate the role of CD200 in intestinal inflammation, we analyzed CD200R expression in immune cells infiltrating inflamed tissues. CD200R is predominantly expressed on myeloid cells, thus we compared its expression in myeloid cells isolated from DSS-treated and control colons and spleens. DSS administration led to diminished expression of CD200R (MFI 3040 ± 220 vs. control 5611 ± 433) in colonic myeloid cells (*p* < 0.001, *t*-test) (Figure 2D), although myeloid cells still had higher levels of CD200R than non-myeloid cells (CD11b^−^CD11c^−^) (Figure 2E). These results indicate that myeloid cells specifically recruited during the development of intestinal inflammation and that CD200R expression decreases during inflammation.

**Figure 3:**
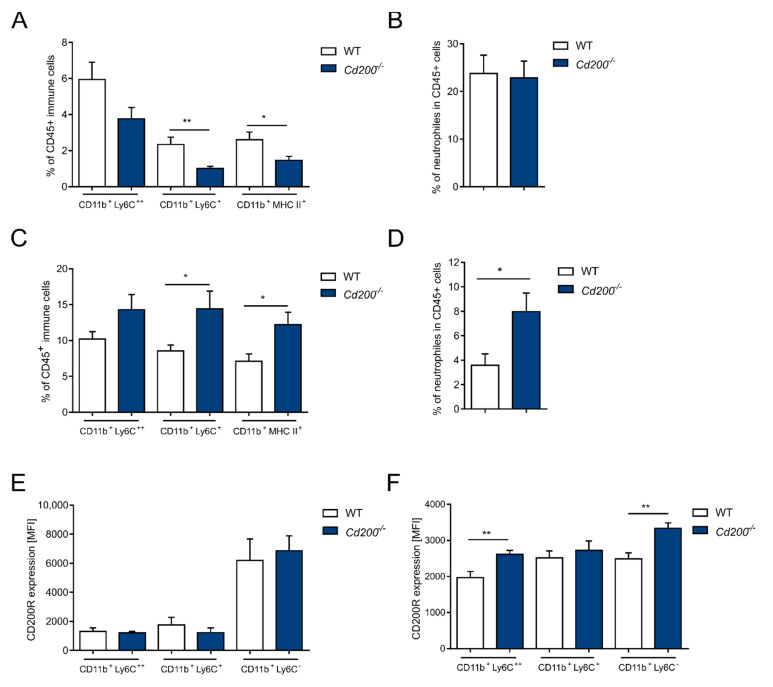
Characterization of myeloid populations in blood and colons of WT and *Cd200^−/−^* mice during DSS-mediated intestinal inflammation. (**A**) Frequency of monocytes (CD11b^+^Ly6C^++^, CD11b^+^Ly6C^+^), macrophages (CD11b^+^CD11c^−^MHC II), and neutrophils (CD11b^+^Ly6G^+^) (**B**) amongst live CD45^+^ cells in blood from DSS-treated WT and *Cd200^−/−^* mice. A. n = 19, B. n = 13–14; (**C**) Percentage of monocytes (CD11b^+^Ly6C^++^ and CD11b^+^Ly6C^+^), macrophages (CD11b^+^CD11c^−^MHC-II^+^) and neutrophils (CD11b^+^Ly6G^+^) (**D**) within the live CD45^+^ cells in colons from DSS-treated WT and *Cd200^−/−^* mice; C. n = 7–10, D. n = 12–15; Expression of CD200R in the particular immune cell populations in the blood (**E**) and colons (**F**) of DSS-treated and *Cd200^−/−^* WT mice. E. n = 6–7, F. n = 6. Results are expressed as mean ± SEM. Significant differences were calculated using Student’s *t*-test * *p* < 0.05. ** *p* < 0.01. compared with WT. MFI: mean fluorescence intensity.

We did not observe differences in the frequency of splenic and colonic immune infiltration between untreated WT and *Cd200^−/−^* mice (Figure S1). To explore whether a lack of CD200 influences the circulation of myeloid cells during intestinal inflammation, we examined the frequency of monocytes, macrophages, and neutrophils in the blood and colon of DSS-treated WT and *Cd200^−/−^* mice. Upon inflammatory conditions, the percentage of blood non-classical monocytes (CD11b^+^Ly6C^+^) in *Cd200^−/−^* mice (1.0 ± 0.1%) was reduced compared to WT mice (2.3 ± 0.4%) (*p* < 0.05, *t*-test) and percentage of macrophages (CD11b^+^MHC II^+^) in *Cd200^−/−^* mice (1.5 ± 0.2%) was reduced compared to WT mice (2.6 ± 0.4%) (*p* < 0.01, *t*-test) and (Figure 3A). The percentage of blood neutrophils did not differ between WT and *Cd200^−/−^* mice (Figure 3B).

The reverse trend in cell composition was observed in the colons, where the percentage of myeloid cells was increased in *Cd200^−/−^* mice. The percentage of non-classical monocytes (CD11b^+^Ly6C^+^) was higher in *Cd200^−/−^* mice than in WT mice (14.4 ± 2.4% vs. 8.5 ± 0.7%) (*p* < 0.05, *t*-test). Similarly, the percentage of macrophages (CD11b^+^MHC II^+^) was higher in *Cd200^−/−^* mice (12.2 ± 1.7%) than in WT mice (7.1 ± 0.9%) (*p* < 0.05, *t*-test) (Figure 3C). In contrast to blood, in colon the percentage of neutrophils was higher in *Cd200^−/−^* mice (7.9 ± 1.5% vs. 3.5 ± 0.9% in WT mice) (*p* < 0.05, *t*-test) (Figure 3D). Blood monocyte subsets had a similar phenotype in both WT and *Cd200^−/−^* mice (data not shown). Inflammatory blood monocytes (CD11b^+^Ly6C^++^ and Ly6C^+^) expressed lower levels of CD200R in comparison to patrolling monocytes (CD11b^+^Ly6C^−^) (Figure 3E). Whereas, in the colon the expression levels of CD200R in Ly6C^+^ and Ly6C^−^ myeloid cell populations were comparable and even further increased in *Cd200^−/−^* mice (Figure 3F).

### 2.3. CD200 Is Expressed on Endothelial Cells and Is Decreased by DSS Administration

**Figure 4:**
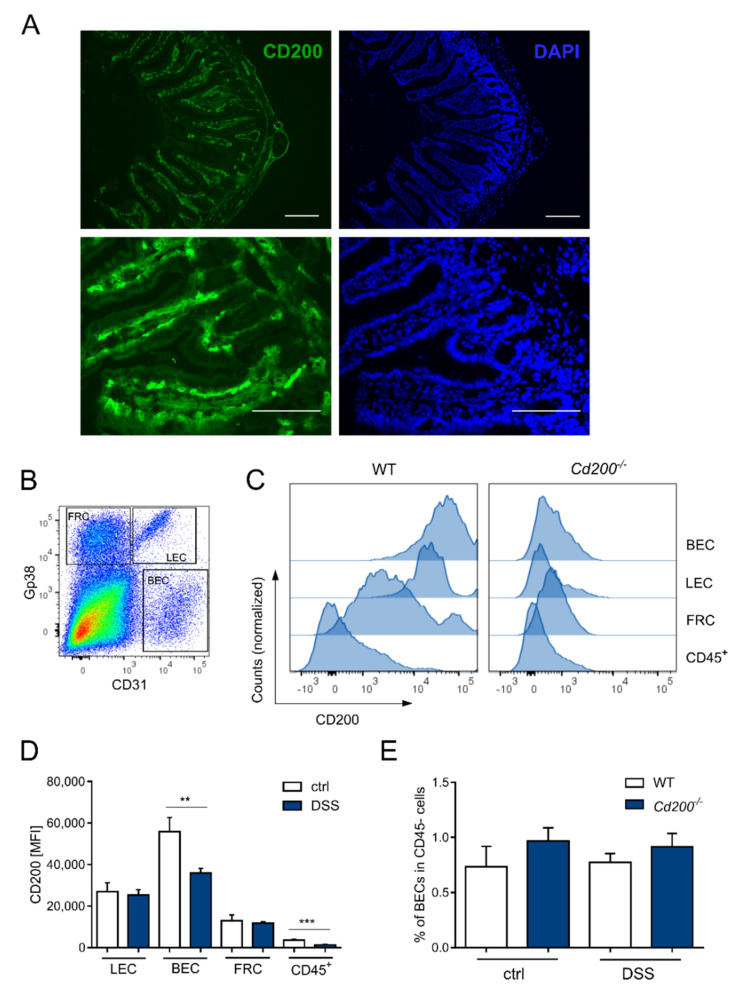
CD200 expression within the intestine. (**A**) Immunofluorescence staining for CD200 (green) in colon tissue from untreated mice. Cell nuclei were visualized by DAPI (blue). Intense CD200 immunostaining was observed in the subepithelial layer of the colonic villi (magnification 40× or 20×), scale bar = 100 µm; (**B**) Example of scatter plot used to identify BEC, LEC, and FRC subsets among CD45^−^ fraction according to Gp38 and CD31 expression; (**C**) Expression of CD200 molecule in BEC, LEC, FRC, and CD45^+^ immune cells in intestines under DSS-induced inflammation; (**D**) CD200 expression in LEC, BEC, FRC, and CD45^+^ cells in colons of WT mice treated with DSS or controls, n = 3–8; (**E**) Frequency of BEC amongst CD45^−^ fraction in the healthy or inflamed colon of WT and *Cd200^−/−^* mice, n = 3–8. Results are expressed as mean ± SEM. Significant differences were calculated using Student’s *t*-test ** *p* < 0.01. *** *p* < 0.001. BEC: blood endothelial cells, LEC: lymphatic endothelial cells, FRC: fibroblastic reticular cells.

To further investigate the role of CD200-CD200R signaling in the intestine, we examined the distribution of one of the ligands for CD200R, CD200. Immunohistochemistry showed intense CD200 immunostaining in the subepithelial part of the colonic villi, covered by CD200 negative epithelium layer (Figure 4A). We also measured expression of three genes that code proteins involved in CD200R signaling: SHIP1, p120RasGAP and Dok2. Gene expression analysis from untreated colons of WT and Cd200^−/−^ mice did not find significant differences between both strains (Figure S2). Next, we confirmed immunohistological results and previous results [26], that high expression of CD200 is characteristic for cells of non-hematopoietic origin, especially blood endothelial cells (BECs, CD31^+^Gp38^−^) and lymphatic endothelial cells (LECs, CD31^+^Gp38^+^), whereas fibroblastic reticular cells (FRCs) had an intermediate CD200 expression (Figure 4B,C). Immune cells (CD45^+^) had the lowest level of CD200 expression (Figure 4C). Interestingly, DSS administration reduced expression of CD200 on BECs (MFI 35910 ± 2269 vs. 55890 ± 6821 in untreated control mice, *p* < 0.01, *t*-test) and CD45^+^ immune cells (MFI 1296 ± 156 vs. 3614 ± 391 in untreated controls, *p* < 0.001, *t*-test). High CD200 expression remained unchanged under inflammatory conditions in FRCs and LECs (Figure 4D). No changes were found in the frequency of BECs and LECs between WT and *Cd200^−/−^* mice, irrespective of DSS administration (Figure 4E). These data suggest that the main source of CD200 in mouse colon are LECs and BECs.

**Figure 5:**
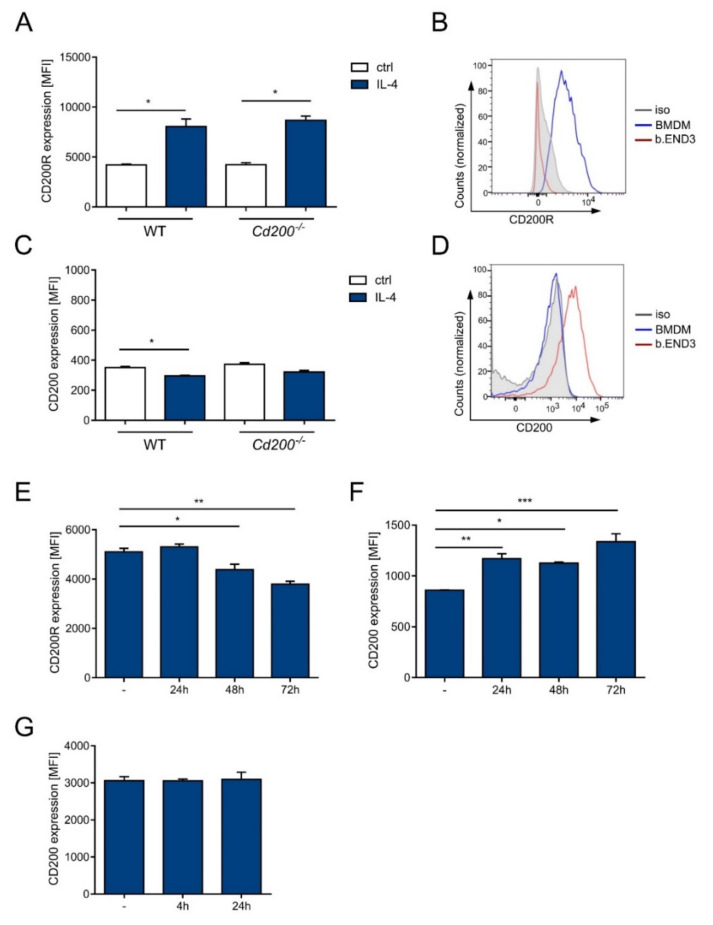
Expression of CD200R and CD200 in BMDMs under IL-4 or LPS stimulation. (**A**) Expression of CD200R in BMDMs isolated from WT and *Cd200^−/−^* mice with or without IL-4 stimulation (20 ng/mL for 24 h), n = 2; (**B**) Exemplary histogram showing expression of CD200R in b.END3 cells and BMDMs are stained with anti-CD200R or isotype control antibody; (**C**) Expression of CD200 in BMDMs isolated from WT and *Cd200^−/−^* mice with or without IL-4 stimulation (20 ng/mL for 24 h) n = 2; (**D**) Exemplary histogram showing expression of CD200 in b.END3 cells and BMDMs are stained with anti-CD200 or isotype control antibody. Expression of CD200R (**E**) and CD200 (**F**) in BMDMs with or without LPS stimulation (100ng/mL) for the indicated time, n = 3; (**G**) Expression of CD200R in b.END3 cells with or without LPS stimulation (100 ng/mL) for the indicated time, n = 2–3. Results are expressed as mean ± SEM, Significant differences were calculated using Student’s *t*-test (panels A, C) or one-way ANOVA (panels E, F) * *p* < 0.05, ** *p* < 0.01, *** *p* < 0.001.

BMDMs from WT and *Cd200^−/−^* mice had high CD200R expression (Figure 5A,B). The level of CD200R was upregulated upon IL-4 treatment to a similar extent in WT and *Cd200^−/−^* mice (in WT MFI 4216 ± 81 vs. 8051 ± 761; in *Cd200^−/−^* 4234 ± 212 vs. 8676 ± 428, *p* < 0.05, *t*-test) (Figure 5A). Conversely, CD200 expression was almost absent in unstimulated or IL-4 stimulated BMDMs (Figure 5C,D). To provide a positive control for CD200^+^ cells, we used b.END3 cells, that lack CD200R but have substantial CD200 expression (Figure 5B,D). LPS treatment of BMDMs mildly downregulated CD200R expression (basic MFI 5100 ± 141 vs. 3792 ± 113 after 72 h-stimulation, *p* < 0.01, one-way ANOVA) (Figure 5E). On the contrary, it induced slight upregulation of CD200 expression in BMDMs (basic MFI 859 ± 2 vs. 1337 ± 77 after 72 h-stimulation, *p* < 0.001, one-way ANOVA) (Figure 5F). CD200 expression in b.END3 cells (basic MFI 3059 ± 109) was higher than in BMDMs (Figure 5F,G). and it was unaffected by LPS treatment (Figure 5G).

### 2.4. Endothelial CD200 Triggers CD200R and Inhibits TNF-α Secretion in BMDMs But Not in the Inflamed Intestine

**Figure 6:**
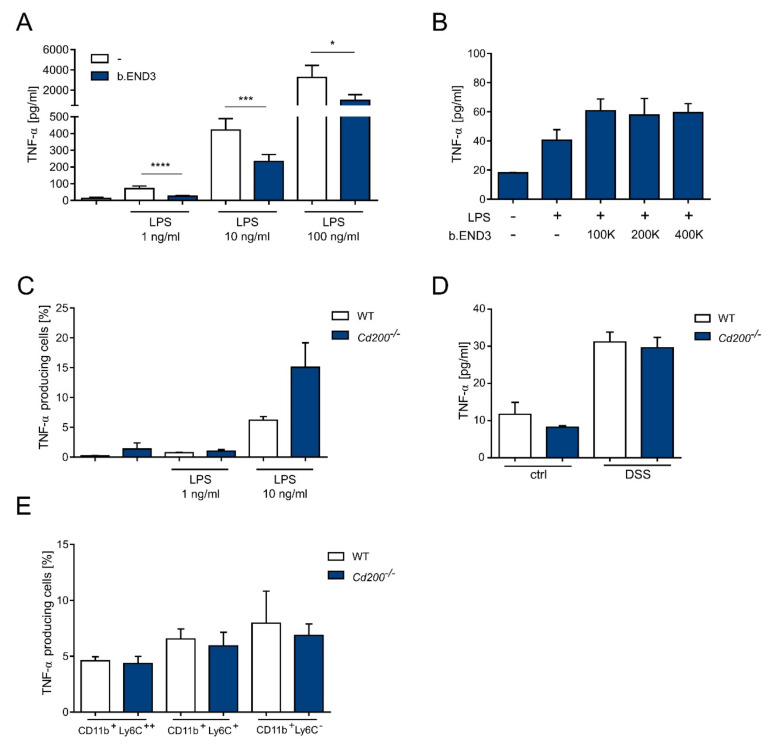
Production of TNF-α by BMDMs and colon tissue in the presence or absence of CD200 expression. (**A**) The concentration of TNF-α was measured in the supernatants from the co-culture of WT BMDMs and b.END3 cells after 24 h of incubation with or without 1–100 ng/mL LPS, n = 4–6; (**B**) The concentration of TNF-α was measured in the supernatants from 24-h co-cultures of WT BMDMs and indicated a number of b.END3 cells with or without 1 ng/mL of LPS without direct contact of two populations (Transwell system), n = 2–3; (**C**) Percentage of TNF-α^+^ WT or *Cd200^−/−^* BMDMs after 4 h incubation with 1 or 10 ng/mL LPS, n = 3. (**D**) The concentration of TNF-α in medium from 24 h culture of colon explants isolated from control or DSS-treated WT and *Cd200^−/−^* mice, n = 8–13; (**E**) Percentage of TNF-α^+^ myeloid cells: CD11b^+^Ly6C^++^, CD11b^+^Ly6C^+^ and CD11b^+^Ly6C^−^ in colons of DSS-treated WT and *Cd200^−/−^* mice, n = 6–9. Results are expressed as mean ± SEM. Significant differences were calculated using Student’s *t*-test * *p* < 0.05. *** *p* < 0.001. **** *p* < 0.0001.

To investigate if CD200, present on the endothelial cells, can influence the activity of BMDMs, we co-cultured b.END3 cells with BMDMs. This experimental setup reflects the potential in vivo interaction between intestinal macrophages and CD200^+^ endothelial cells. For the co-cultures with b.END3 cells, BMDM were isolated from Balb/c mice to keep a syngeneic setup of cells in the experiment. Cells were stimulated with a concentration range of LPS (1–100 ng/mL). We observed a dose-dependent secretion of TNF-α, which was suppressed by b.END3 cells from 71.1 ± 6 pg/mL to 25.1 ± 1.6 pg/mL at 1 ng/mL of LPS (*p* < 0.0001, *t*-test) and from 3256 ± 533 pg/mL to 1007 ± 274 pg/mL at 100 ng/mL of LPS (*p* < 0.05, *t*-test) (Figure 6A). Importantly, when BMDMs and b.END3 were co-cultured without direct contact (Transwell setup), we did not observe inhibition of TNF-α production by b.END3, whereas the presence of b.END3 separated from direct contact with BMDMs did not significantly increase TNF-α production (Figure 6B). We also measured TNF-α production in BMDMs isolated from WT and *Cd200^−/−^* mice. Increasing LPS concentrations increased the fraction of TNF-α-producing cells. However, there was no significant difference between cells isolated from WT and *Cd200^−/−^* mice (Figure 6C) suggesting that low expression of CD200 in BMDMs was not sufficient to inhibit TNF-α production upon LPS stimulation. Additionally, we checked expression of four additional cytokines including IL-1α, IL-1β, IL-6, IL-10 in BMDMs isolated from WT and *Cd200^−/−^* mice. We found that upon stimulation with LPS (1–10 ng/mL LPS) these cytokines were similarly expressed in cells derived from WT and *Cd200^−/−^* mice (Figure S3). To investigate the mechanistic relationship of CD200R with intestinal inflammation, we measured TNF-α production in ex vivo cultures of colon explants. Tissues isolated from DSS-treated mice were cultured, without additional stimulation, and the concentration of TNF-α was measured in the medium. There was no difference in TNF secretion by colons isolated from WT and *Cd200^−/−^* mice neither in control (11.7 ± 3.1 pg/mL vs. 8.2 ± 0.4 pg/mL) nor DSS-treated mice (31.1 ± 2.6 pg/mL vs. 29.6 ± 2.7 pg/mL) (Figure 6D). Similarly, we did not find significant differences between WT and *Cd200^−/−^* colons in IL-6 production, another proinflammatory cytokine (Figure S4). TNF-α production was also analyzed by flow cytometry in three major myeloid cell populations in the colon: CD11b^+^Ly6C^++^, CD11b^+^Ly6C^+^, CD11b^+^Ly6C^−^, isolated from WT and *Cd200^−/−^* mice. Under inflammatory conditions, up to 10% of CD11b^+^ cells in the colon produced TNF-α with no significant difference in the percentage of TNF-α-positive myeloid cells between WT and *Cd200^−/−^* mice (Figure 6E).

The above results suggest that although CD200-CD200R interaction can regulate TNF-α production, in inflamed colons, despite the presence of CD200 expression, there is no difference in TNF-α production between WT and *Cd200^−/−^* mice.

## 3. Discussions

The inhibitory receptor CD200R is highly expressed on myeloid cells in tissues frequently exposed to pathogens such as the lung, where increased immune tolerance to innocuous antigens must be tightly regulated to prevent inflammatory disorders [35,36]. The gastrointestinal tract is another example of tissue continuously exposed to fungal, viral, and bacterial presence. The anti-inflammatory properties of resident intestinal macrophages, which contribute to intestinal homeostasis may be regulated by CD200R.

The first investigation of CD200R and CD200 importance during intestinal inflammation was performed by Bain et al. in the SPF animal facility [37]. They showed that intestinal inflammation of *Cd200R^−/−^* mice was not different from controls. Importantly, they found that weight loss was reduced transiently in *Cd200^−/−^* mice than in controls. What is in line with our findings. Also, disease score and colon length described by Bain et al. were less severe in *Cd200^−/−^* mice, but these two parameters were not significantly different. Conversely, Chen et al. also using SPF-maintained mice showed that *Cd200^−/−^* and *Cd200R^−/−^* display more severe colitis with accelerated infiltration of macrophages and monocytes and greater expression of pro-inflammatory cytokines [38]. These observations are in line with a general observation showing increased susceptibility of mice with inhibited CD200-CD200R pathway to the increased pro-inflammatory activity of myeloid cells [20,29,35,39]. However there are also findings showing the anti-inflammatory effect of CD200 in the regulation of IL-10 [40].

In our study, in conventionally reared mice, we observed a greater infiltration of monocytes, macrophages, and neutrophils in the colon of *Cd200^−/−^* mice after DSS administration. Mice housed in the conventional animal facility and exposed to a wider spectrum of commensal microbiota, displayed moderate signs of intestinal inflammation when administrated with DSS. In comparison to WT mice, *Cd200^−/−^* mice exhibited smaller histological changes within the colon and decreased histological score (DAI). This was accompanied by a decreased percentage of monocytes and macrophages in colons *Cd200^−/−^* mice in comparison to WT controls. A possible explanation for these differences is the composition of microbiota present in mice reared in different animal facilities. Possibly conventionally-reared mice, in contrast to SPF-reared mice, might have a microbiota that reverses the effect of the lack of CD200 expression in the intestine. Local depletion of CD200R-positive macrophages would further address their role in intestinal inflammation performed in SPF versus conventional conditions.

CD200^+^ mesenchymal stem cells can suppress TNF-α secretion by macrophage-like THP-1 cells [41]. Here we showed CD200^+^ endothelial cells inhibit the production of TNF-α by primary BMDMs stimulated with bacterial LPS. Our results indicate that there is an abundance of CD200 ligand in the intestinal tissue, expressed predominantly on non-immune cells. However, during DSS-induced inflammation complete lack of CD200 expression does not affect the total production of TNF-α in the colon, suggesting that other mechanisms are involved in the observed effect in *Cd200^−/−^* mice. Interestingly, two new functional CD200R ligands have been identified in mouse intestine iSEC1 and iSEC2. Both proteins are expressed by secretory epithelial cells and can diminish the activation of intestinal intraepithelial lymphocytes [27]. Still, the expression of these alternative agonists of CD200R does not explain the ameliorated inflammation present in *Cd200^−/−^* mice.

Obtained results of conventionally reared *Cd200^−/−^* mice that have diminished intestinal inflammation suggest that the composition of intestinal microbiota affects the CD200-dependent regulation of the immune response. The inhibitory signaling pathways, including the CD200-CD200R axis, have been exploited by microbes to suppress host defenses. Several species of bacteria and viruses express proteins that mimic the CD200 molecule and are able to trigger CD200R, reviewed by Vane et al. [42]. In this case, the induction of the CD200-CD200R axis is like a double-edged sword, which protects the host from excessive inflammation in response to pathogens [43] and allows pathogens to survive and live at the expense of the host [44]. Neisseria meningitides induces differentiation of monocytes into CD200R-positive macrophages to maintain asymptomatic colonization in human mucosa [45]. A shift of pro-inflammatory macrophages to anti-inflammatory macrophages was shown to alleviate experimental colitis in mice and this strategy could be beneficial for IBD patients [46]. Grainger et al. have observed that inflammatory monocytes acquire a regulatory phenotype during acute gastrointestinal infection of mice with Toxoplasma gondii, and inhibit neutrophils via the production of prostaglandin E2 [47]. Interestingly, the observed effect was strictly dependent on the presence of commensals since in germ-free mice production of immunoregulatory cytokines was compromised. Lack of intestinal microbiota reduces IL-10 production by resident macrophages and restores susceptibility for LPS-stimulation [47], indicating a great capacity of microbiota to trigger regulatory mechanisms in myeloid cells during inflammation. Recently, Horuluoglu et al. found that the bacterial product Pam3CSK4 modifies immune responses, and the outcome of intestinal inflammation by promoting differentiation of alternatively activated macrophages [18]. It was also shown that CD200R is a hallmark of macrophages with the anti-inflammatory function. Nevertheless, the expression of CD200 did not further induce suppression of macrophages, as was suggested elsewhere [48,49] since DSS-treated WT mice displayed more severe inflammation than mice without CD200.

The anti-inflammatory properties of intestine macrophages depend on IL-10 and TGF-β secretion in colonic mucosa [50] and CD200R stimulation can suppress activity and IL-10 production of anti-inflammatory tumor-associated myeloid cells [40]. Thus, lack of CD200 may increase the suppressive activity of myeloid cells during colitis, which may explain why we see ameliorated colitis in *Cd200^−/−^* mice.

In conclusion, although CD200 is highly expressed in the intestine and potentially able to inhibit inflammatory activation of myeloid cells, lack of this molecule does not change the expression of TNF-α in the colon during DSS-induced inflammation. Whereas, the lack of CD200 expression does not increase the severity of intestinal inflammation in conventionally-reared mice. This result indicates that the intestinal microbiota may have an unexpected influence on CD200R-dependent immune regulation. What suggests that the importance of microbiota should be taken into consideration when choosing the immunoregulatory therapies to treat inflammation in the non-sterile tissue.

## 4. Materials and Methods

### 4.1. Animals

Wild-type (WT) and *Cd200^−/−^* mice on a C57Bl/6 background were obtained originally from UMC Utrecht, The Netherlands. Mice were housed under conventional conditions at the Maria Sklodowska-Curie Institute of Oncology (Warsaw, Poland). For the experiments, all mice (only females) were housed in the conventional facilities of Medical University of Warsaw. Mice were supplied with regular laboratory chow and water ad libitum and were used between 6 and 12 weeks of age. Donor mice used for bone marrow macrophages isolation were purchased from the Animal Facility of Medical University of Warsaw (C57Bl/6) or The Center for Experimental Medicine of the Medical University of Bialystok (BALB/c) and were housed under SPF conditions at the Animal Facility of the Medical University of Warsaw and were typically 6 to 8 weeks old. All animal experiments were performed in accordance with the national regulations and were approved by the Local Ethics Committee, Warsaw, Poland.

### 4.2. DSS-Induced Colitis

Acute colitis was induced by administrating mice 3% dextran sodium sulfate (DSS; reagent grade; MW 36,000–50,000 kDa; MP Biomedicals,) dissolved in drinking water for 6 days. Mice were monitored for weight loss daily and at the end of the experiment disease activity index (DAI) was generated using the following parameters: weight loss (0–4), colon length (0–4), stool consistency (0–2), stool blood (0–1), rectal bleeding (0–1). Animals were sacrificed on day 8 and the colon, appendix, spleen, and blood were isolated for further analysis.

### 4.3. Histological Analysis of Colon Samples

Colons were removed and washed with cold PBS. For immunofluorescent staining, the intestines of wild type untreated mice were washed and snap-frozen on dry ice. Sections were stained with anti-CD200 (OX-90) rat monoclonal antibody (Serotec, part of Bio-Rad, Hercules, CA, USA), followed by secondary anti-Rat IgG-Alexa488 antibody and subsequent DAPI (Sigma Aldrich, Saint Louis, MO, USA). For the general histology, colons were fixed in 10% neutral, buffered formalin solution. Paraffin-embedded samples were cut to 5 μm sections and stained with Hematoxilin-Eosin (HE) according to standard protocol. Images were digitalized using slide scan Axio Scan.Z1 (ZEISS) and ZEN 2 imaging software.

Samples were blind-scored according to Cooper et al. [51], which is routinely used for histological scoring of IBD severity. Colon damage was evaluated taking into account the epithelial erosion and the inflammatory cell infiltration, as reported in Table 1. Briefly, mucosal damage was scored using a numerical scale: from the normal epithelium (0 points) to ulceration (6 points) considered the worst lesion, while a three-point score was adopted for the histological evaluation of colon inflammation respectively for inflammation in the mucosal, submucosal and muscular/serosal layers. From each parameter, the sum of the points was defined as the mucosal damage/inflammation scores.

### 4.4. Isolation of Colonic Lamina Propria Cells

The colons and appendixes were excised and washed with cold PBS and opened lengthwise, washed with fresh cold PBS, and cut into small strips (~0.4 cm). Next, tissue fragments were mixed slowly in ice-cold 5 mM EDTA in PBS for 15 min and then vortexed to remove epithelial cells. Tissue fragments were then digested using 400 U/mL collagenase (Sigma C5138, from Clostridium histolyticum, Type IV, 0.5–5.0 FALGPA units/mg solid, ≥125 CDU/mg solid) and 600 U/mL DNAse (Sigma DN25 ≥ 400 Kunitz units/mg) solution in 2 mL of complete RPMI 1640 (Sigma) containing 100 units/mL of penicillin and 100 µg/mL of streptomycin (Sigma), and 10% FBS (HyClone laboratories, Logan, UT, USA) for 60 min at 37 °C with gentle mixing. Next, samples were dissociated using a gentle MACS dissociator (Miltenyi Biotec), passed through a 70 µm cell strainer (BD Falcon), and washed with PBS. After centrifugation (500× *g*) lamina propria cells were collected and kept on ice until further use.

### 4.5. Preparation of Splenocytes and Blood Cells

To obtain single-cell suspension, the spleen was forced through a 100 µm cell strainer (BD Falcon) using a syringe piston and washed with PBS. To remove red blood cells, 5 mL ACK Lysis Buffer (Gibco) was added, and cells were incubated for 5 min at room temperature. Cells were then washed and resuspended in PBS. Peripheral blood was isolated from heart puncture, immediately after euthanasia. Blood samples were collected to tubes containing EDTA anticoagulant (Microvette 500 μL, SARSTED) and kept on ice until use. Erythrocytes from a blood sample (50 µL) were lysed using 10 mL ACK Lysis Buffer (Gibco) in the same way as splenocytes. Cells were kept on ice until further use.

### 4.6. Flow Cytometric Analysis

Single-cell suspensions prepared from blood, spleen, and colon were pre-incubated with 5% rat serum for 20 min to inhibit non-specific binding of rat antibodies. Cells were then incubated for 30 min at 4 °C with fluorescently labelled monoclonal antibodies: V500 anti-mouse CD45.2 (clone 104), 605NC anti-mouse CD11b (clone M1/70), PerCP-Cy5 anti-mouse CD8 (clone 53–6.7) from BD Biosciences; PE anti-mouse CD200R (clone OX110), PE anti-mouse CD200 (clone OX90), Alexa 488 anti-mouse CD11b (clone M1/70), PE-Cy7 anti-mouse F4/80 (clone BM8), eFluor^®^ 450 anti-mouse CD11c (clone N418;), APC anti-mouse Ly6C (clone HK1.4), PE-Cy7 anti-mouse MHC II (clone M5/114.15.2), FITC anti-mouse CD4 (clone L3T4,) from eBioscience; PerCP-Cy5 anti-mouse Gr-1 (clone RB6-8C5; BD Pharmingen); B220 PE anti-mouse CX3CR1 (clone SA011F11; BioLegend); Alexa 700 anti-mouse CCR2 (clone 475301; R&D systems). Cells were then washed, resuspended in Flow Cytometry Staining Buffer (BD), and analyzed. Before intracellular staining of cytokines, isolated cells were re-stimulated, for 4 h at 37 °C, with the indicated dose of LPS (Invivogen) in the presence of the intracellular protein transport inhibitor BD GolgiStop™ (BD) (1:1000 dilution). Samples were then allowed for fixation followed by permeabilization according to the manufacturer’s recommended protocol (eBioscience) with slight modifications. In brief, extracellular markers were stained as described above. After the last wash, pellets were resuspended in 100 μL of IC Fixation Buffer (eBioscience) and incubated in the dark at 4 °C overnight. Samples were permeabilized by Permeabilization Buffer (eBioscience) and stained using PE anti-mouse TNF-α antibody (clone MP6-XT22, eBiosciences) in 100 µL 1X Permeabilization Buffer and incubated in the dark at room temperature for 40 min. Cells were washed with 1X Permeabilization Buffer, centrifuged, and resuspended in Flow Cytometry Staining Buffer for analysis. All samples were analyzed on the BD FACSAria or BD FACSCanto and analysis was performed using FACS Diva or FlowJow software.

### 4.7. L929 Culture and Generation of CM

To obtain L929-conditioned medium (CM), L929 cells were seeded into T-125 flasks in 50 mL of culture medium DMEM/F-12 (Gibco, part of Thermo Fisher, Waltham, MA, USA) supplemented with 10% FBS (HyClone), 1% pen-strep (Sigma Aldrich, Saint Louis, MO, USA), and cultured until full confluency. Next, the medium was changed and cells were cultured for an additional 3 days. At that point, the medium was collected, filtered through a 0.22 μm filter, and stored in aliquots at −80 °C.

### 4.8. BMDM Generation

BALB/c or C57Bl/6 mice were euthanized by rapid cervical dislocation, femurs were gently isolated and washed with PBS. Bone marrow cells were flushed out of the bones, washed twice with PBS. Erythrocytes were lysed using ACK Lysis Buffer (Gibco part of Thermo Fisher, Waltham, MA, USA), similarly as in the case of splenocytes. Next, cells were counted and plated on a sterile Petri dish (2 × 10^5^ cells/mL) in 10 mL of macrophage complete medium DMEM/F-12 (Gibco), supplemented with 10% FBS (HyClone), 1% pen-strep (Sigma Aldrich) and 20% L929 CM. The fresh full macrophage medium was changed every 3 days. When indicated, BMDMs were stimulated with lipopolysaccharide (LPS) or IL-4 (Invivogen, San Diego, CA, USA) for 4, 24, 48, or 72 h. Next, cells were detached and stained for flow cytometry.

### 4.9. TNF-α and IL-6 Analysis

The b.END3 cells (from ATCC) were maintained in DMEM culture medium (Corning) containing 10% FBS and 1% penicillin/streptomycin. For co-culture experiments, 5-day old bone marrow-derived macrophages (BMDMs) were seeded (1 × 10^5^ cells/mL) on 12-well plates. On day 6 b.END3 cells were seeded on top of the BMDM culture and further incubated for 24 h. For co-culture without direct contact, cells were seeded into plates with Transwell chambers: BMDMs were seeded on the bottom and b.END3 cells were seeded in the insert. When indicated, cells in monocultures or co-cultures were treated with LPS (Invivogen) for 4, 24, 48, or 72 h and analyzed by flow cytometry. Alternatively, the concentration of TNF-α in the supernatants was measured by enzyme-linked immunosorbent assay (ELISA).

Colon tissue fragments (5 mm long) from the distal part were cultured in 200 µL of RPMI-1640 (Sigma Aldrich) supplemented with 10% FBS (HyClone), 1% pen-strep (Sigma Aldrich) for 24 h at 37 °C. The concentration of TNF-α was measured in culture supernatants by ELISA (eBioscience or Invitrogen part of Thermo Fisher, Waltham, MA, USA). ELISA was performed according to the manufacturer’s protocol, with detection done using Asys UVM 340 Microplate Reader (Biochrom, Cambridge, UK).

### 4.10. Statistical Analysis

All results are shown as mean ± standard error of the mean. Data were analyzed using GraphPad Prism v7.03 software (GraphPad, La Jolla, CA, USA). One-way ANOVA test was used for the analysis of CD200R and CD200 expression upon several stimulations and Student’s *t*-test was used for all other comparisons. A *p* value < 0.05 was considered statistically significant.

## Figures and Tables

**Table 1:** Histological score of colitis.

**A. Grade of Epithelial Damage**	**Score**
Normal	0
Hyperproliferation, irregular crypts, goblet cell loss	1
Slight to mild crypt loss (10–50%)	2
Severe crypt loss (50–90%)	3
Complete crypt loss	4
Mucosal erosion/small sized ulcer (<5 crypt widths)	5
Medium -to large ulcer (>5 crypt widths)	6
**B. Grade of inflammation**	**Score**
Normal	0
Slight	1
Mild	2
Severe	3

## Data Availability

Not applicable.

## Supplementary Materials

The following are available online at https://www.mdpi.com/article/10.3390/ijms22105358/s1.

## References

[1] Zhang Y.-Z., Li Y.-Y. (2014). Inflammatory Bowel Disease: Pathogenesis. World J. Gastroenterol. WJG.

[2] Murch S.H., Braegger C.P., Walker-Smith J.A., MacDonald T.T. (1993). Location of Tumour Necrosis Factor Alpha by Immunohistochemistry in Chronic Inflammatory Bowel Disease. Gut.

[3] Baumann H., Gauldie J. (1994). The Acute Phase Response. Immunol. Today.

[4] Reimund J.M., Wittersheim C., Dumont S., Muller C.D., Baumann R., Poindron P., Duclos B. (1996). Mucosal Inflammatory Cytokine Production by Intestinal Biopsies in Patients with Ulcerative Colitis and Crohn’s Disease. J. Clin. Immunol..

[5] Olsen T., Goll R., Cui G., Husebekk A., Vonen B., Birketvedt G.S., Florholmen J. (2007). Tissue Levels of Tumor Necrosis Factor-Alpha Correlates with Grade of Inflammation in Untreated Ulcerative Colitis. Scand. J. Gastroenterol..

[6] Begue B., Wajant H., Bambou J.-C., Dubuquoy L., Siegmund D., Beaulieu J.-F., Canioni D., Berrebi D., Brousse N., Desreumaux P. (2006). Implication of TNF-Related Apoptosis-Inducing Ligand in Inflammatory Intestinal Epithelial Lesions. Gastroenterology.

[7] Platt A.M., Bain C.C., Bordon Y., Sester D.P., Mowat A.M. (2010). An Independent Subset of TLR Expressing CCR2-Dependent Macrophages Promotes Colonic Inflammation. J. Immunol..

[8] Hadis U., Wahl B., Schulz O., Hardtke-Wolenski M., Schippers A., Wagner N., Müller W., Sparwasser T., Förster R., Pabst O. (2011). Intestinal Tolerance Requires Gut Homing and Expansion of FoxP3+ Regulatory T Cells in the Lamina Propria. Immunity.

[9] Bain C.C., Scott C.L., Uronen-Hansson H., Gudjonsson S., Jansson O., Grip O., Guilliams M., Malissen B., Agace W.W., Mowat A.M. (2013). Resident and Pro-Inflammatory Macrophages in the Colon Represent Alternative Context-Dependent Fates of the Same Ly6Chi Monocyte Precursors. Mucosal Immunol..

[10] Smythies L.E., Shen R., Bimczok D., Novak L., Clements R.H., Eckhoff D.E., Bouchard P., George M.D., Hu W.K., Dandekar S. (2010). Inflammation Anergy in Human Intestinal Macrophages Is Due to Smad-Induced IkappaBalpha Expression and NF-KappaB Inactivation. J. Biol. Chem..

[11] Bain C.C., Bravo-Blas A., Scott C.L., Perdiguero E.G., Geissmann F., Henri S., Malissen B., Osborne L.C., Artis D., Mowat A.M. (2014). Constant Replenishment from Circulating Monocytes Maintains the Macrophage Pool in the Intestine of Adult Mice. Nat. Immunol..

[12] Kamada N., Hisamatsu T., Okamoto S., Chinen H., Kobayashi T., Sato T., Sakuraba A., Kitazume M.T., Sugita A., Koganei K. (2008). Unique CD14 Intestinal Macrophages Contribute to the Pathogenesis of Crohn Disease via IL-23/IFN-Gamma Axis. J. Clin. Investig..

[13] Mowat A.M., Bain C.C. (2011). Mucosal Macrophages in Intestinal Homeostasis and Inflammation. J. Innate Immun..

[14] Thiesen S., Janciauskiene S., Uronen-Hansson H., Agace W., Högerkorp C.-M., Spee P., Håkansson K., Grip O. (2014). CD14(Hi)HLA-DR(Dim) Macrophages, with a Resemblance to Classical Blood Monocytes, Dominate Inflamed Mucosa in Crohn’s Disease. J. Leukoc. Biol..

[15] Du Z., Hudcovic T., Mrazek J., Kozakova H., Srutkova D., Schwarzer M., Tlaskalova-Hogenova H., Kostovcik M., Kverka M. (2015). Development of Gut Inflammation in Mice Colonized with Mucosa-Associated Bacteria from Patients with Ulcerative Colitis. Gut Pathog..

[16] Hans W., Schölmerich J., Gross V., Falk W. (2000). The Role of the Resident Intestinal Flora in Acute and Chronic Dextran Sulfate Sodium-Induced Colitis in Mice. Eur. J. Gastroenterol. Hepatol..

[17] Kitajima S., Morimoto M., Sagara E., Shimizu C., Ikeda Y. (2001). Dextran Sodium Sulfate-Induced Colitis in Germ-Free IQI/Jic Mice. Exp. Anim..

[18] Horuluoglu B.H., Kayraklioglu N., Tross D., Klinman D. (2020). PAM3 Protects against DSS-Induced Colitis by Altering the M2:M1 Ratio. Sci. Rep..

[19] Bain C.C., Mowat A.M. (2014). Macrophages in Intestinal Homeostasis and Inflammation. Immunol. Rev..

[20] Wright G.J., Puklavec M.J., Willis A.C., Hoek R.M., Sedgwick J.D., Brown M.H., Barclay A.N. (2000). Lymphoid/Neuronal Cell Surface OX2 Glycoprotein Recognizes a Novel Receptor on Macrophages Implicated in the Control of Their Function. Immunity.

[21] Wright G.J., Cherwinski H., Foster-Cuevas M., Brooke G., Puklavec M.J., Bigler M., Song Y., Jenmalm M., Gorman D., McClanahan T. (2003). Characterization of the CD200 Receptor Family in Mice and Humans and Their Interactions with CD200. J. Immunol..

[22] Zhang S., Cherwinski H., Sedgwick J.D., Phillips J.H. (2004). Molecular Mechanisms of CD200 Inhibition of Mast Cell Activation. J. Immunol..

[23] Webb M., Barclay A.N. (1984). Localisation of the MRC OX-2 Glycoprotein on the Surfaces of Neurones. J. Neurochem..

[24] Barclay A.N. (1981). Different Reticular Elements in Rat Lymphoid Tissue Identified by Localization of Ia, Thy-1 and MRC OX 2 Antigens. Immunology.

[25] Wright G.J., Jones M., Puklavec M.J., Brown M.H., Barclay A.N. (2001). The Unusual Distribution of the Neuronal/Lymphoid Cell Surface CD200 (OX2) Glycoprotein Is Conserved in Humans. Immunology.

[26] Ko Y.-C., Chien H.-F., Jiang-Shieh Y.-F., Chang C.-Y., Pai M.-H., Huang J.-P., Chen H.-M., Wu C.-H. (2009). Endothelial CD200 Is Heterogeneously Distributed, Regulated and Involved in Immune Cell-Endothelium Interactions. J. Anat..

[27] Kojima T., Tsuchiya K., Ikemizu S., Yoshikawa S., Yamanishi Y., Watanabe M., Karasuyama H. (2016). Novel CD200 Homologues ISEC1 and ISEC2 Are Gastrointestinal Secretory Cell-Specific Ligands of Inhibitory Receptor CD200R. Sci. Rep..

[28] Jenmalm M.C., Cherwinski H., Bowman E.P., Phillips J.H., Sedgwick J.D. (2006). Regulation of Myeloid Cell Function through the CD200 Receptor. J. Immunol..

[29] Copland D.A., Calder C.J., Raveney B.J.E., Nicholson L.B., Phillips J., Cherwinski H., Jenmalm M., Sedgwick J.D., Dick A.D. (2007). Monoclonal Antibody-Mediated CD200 Receptor Signaling Suppresses Macrophage Activation and Tissue Damage in Experimental Autoimmune Uveoretinitis. Am. J. Pathol..

[30] Elshal M.F., Aldahlawi A.M., Saadah O.I., McCoy J.P. (2015). Reduced Dendritic Cells Expressing CD200R1 in Children with Inflammatory Bowel Disease: Correlation with Th17 and Regulatory T Cells. Int. J. Mol. Sci..

[31] Elshal M.F., Aldahlawi A.M., Saadah O.I., Philip McCoy J. (2016). Expression of CD200R1 and Its Ligand CD200 on T-Helper Lymphocytes of Pediatric Patients with Ulcerative Colitis and Crohn’s Disease. Clin. Lab..

[32] Karnam G., Rygiel T.P., Raaben M., Grinwis G.C.M., Coenjaerts F.E., Ressing M.E., Rottier P.J.M., de Haan C.A.M., Meyaard L. (2012). CD200 Receptor Controls Sex-Specific TLR7 Responses to Viral Infection. PLoS Pathog..

[33] Pilch Z., Tonecka K., Braniewska A., Sas Z., Skorzynski M., Boon L., Golab J., Meyaard L., Rygiel T.P. (2018). Antitumor Activity of TLR7 Is Potentiated by CD200R Antibody Leading to Changes in the Tumor Microenvironment. Cancer Immunol. Res..

[34] Perše M., Cerar A. (2012). Dextran Sodium Sulphate Colitis Mouse Model: Traps and Tricks. J. Biomed. Biotechnol..

[35] Snelgrove R.J., Goulding J., Didierlaurent A.M., Lyonga D., Vekaria S., Edwards L., Gwyer E., Sedgwick J.D., Barclay A.N., Hussell T. (2008). A Critical Function for CD200 in Lung Immune Homeostasis and the Severity of Influenza Infection. Nat. Immunol..

[36] Rygiel T.P., Rijkers E.S.K., de Ruiter T., Stolte E.H., van der Valk M., Rimmelzwaan G.F., Boon L., van Loon A.M., Coenjaerts F.E., Hoek R.M. (2009). Lack of CD200 Enhances Pathological T Cell Responses during Influenza Infection. J. Immunol..

[37] Bain C.C., Mowat A.M. (2012). CD200 Receptor and Macrophage Function in the Intestine. Immunobiology.

[38] Chen Z., Yu K., Zhu F., Gorczynski R. (2016). Over-Expression of CD200 Protects Mice from Dextran Sodium Sulfate Induced Colitis. PLoS ONE.

[39] Broderick C., Hoek R.M., Forrester J.V., Liversidge J., Sedgwick J.D., Dick A.D. (2002). Constitutive Retinal CD200 Expression Regulates Resident Microglia and Activation State of Inflammatory Cells during Experimental Autoimmune Uveoretinitis. Am. J. Pathol..

[40] Wang L., Liu J.-Q., Talebian F., El-Omrani H.Y., Khattabi M., Yu L., Bai X.-F. (2010). Tumor Expression of CD200 Inhibits IL-10 Production by Tumor-Associated Myeloid Cells and Prevents Tumor Immune Evasion of CTL Therapy. Eur. J. Immunol..

[41] Pietilä M., Lehtonen S., Tuovinen E., Lähteenmäki K., Laitinen S., Leskelä H.-V., Nätynki A., Pesälä J., Nordström K., Lehenkari P. (2012). CD200 Positive Human Mesenchymal Stem Cells Suppress TNF-Alpha Secretion from CD200 Receptor Positive Macrophage-Like Cells. PLoS ONE.

[42] Vaine C.A., Soberman R.J. (2014). The CD200-CD200R1 Inhibitory Signaling Pathway: Immune Regulation and Host-Pathogen Interactions. Adv. Immunol..

[43] Mukhopadhyay S., Plüddemann A., Hoe J.C., Williams K.J., Varin A., Makepeace K., Aknin M.-L., Bowdish D.M.E., Smale S.T., Barclay A.N. (2010). Immune Inhibitory Ligand CD200 Induction by TLRs and NLRs Limits Macrophage Activation to Protect the Host from Meningococcal Septicemia. Cell Host Microbe.

[44] Cortez M., Huynh C., Fernandes M.C., Kennedy K.A., Aderem A., Andrews N.W. (2011). Leishmania Promotes Its Own Virulence by Inducing Expression of the Host Immune Inhibitory Ligand CD200. Cell Host Microbe.

[45] Wang X., Sjölinder M., Gao Y., Wan Y., Sjölinder H. (2016). Immune Homeostatic Macrophages Programmed by the Bacterial Surface Protein NhhA Potentiate Nasopharyngeal Carriage of Neisseria Meningitidis. MBio.

[46] Zhu Y., Li X., Chen J., Chen T., Shi Z., Lei M., Zhang Y., Bai P., Li Y., Fei X. (2016). The Pentacyclic Triterpene Lupeol Switches M1 Macrophages to M2 and Ameliorates Experimental Inflammatory Bowel Disease. Int. Immunopharmacol..

[47] Grainger J.R., Wohlfert E.A., Fuss I.J., Bouladoux N., Askenase M.H., Legrand F., Koo L.Y., Brenchley J.M., Fraser I.D.C., Belkaid Y. (2013). Inflammatory Monocytes Regulate Pathologic Responses to Commensals during Acute Gastrointestinal Infection. Nat. Med..

[48] Koning N., van Eijk M., Pouwels W., Brouwer M.S.M., Voehringer D., Huitinga I., Hoek R.M., Raes G., Hamann J. (2010). Expression of the Inhibitory CD200 Receptor Is Associated with Alternative Macrophage Activation. J. Innate Immun..

[49] Fallarino F., Asselin-Paturel C., Vacca C., Bianchi R., Gizzi S., Fioretti M.C., Trinchieri G., Grohmann U., Puccetti P. (2004). Murine Plasmacytoid Dendritic Cells Initiate the Immunosuppressive Pathway of Tryptophan Catabolism in Response to CD200 Receptor Engagement. J. Immunol..

[50] Shouval D.S., Biswas A., Goettel J.A., McCann K., Conaway E., Redhu N.S., Mascanfroni I.D., Adham Z.A., Lavoie S., Ibourk M. (2014). Interleukin-10 Receptor Signaling in Innate Immune Cells Regulates Mucosal Immune Tolerance and Anti-Inflammatory Macrophage Function. Immunity.

[51] Cooper H.S., Murthy S.N., Shah R.S., Sedergran D.J. (1993). Clinicopathologic Study of Dextran Sulfate Sodium Experimental Murine Colitis. Lab. Investig. J. Tech. Methods Pathol..

