# Supplementary material for: The CD200 Regulates Inflammation in Mice Independently of TNF-α Production
Source: Int J Mol Sci. 2021 May 19;22(10):5358. doi: 10.3390/ijms22105358 (PMC8161250; doi:10.3390/ijms22105358)
Supplement: Supplementary file 1 [file ijms-22-05358-s001.zip › ijms-1164811-supplementary.pdf]

A

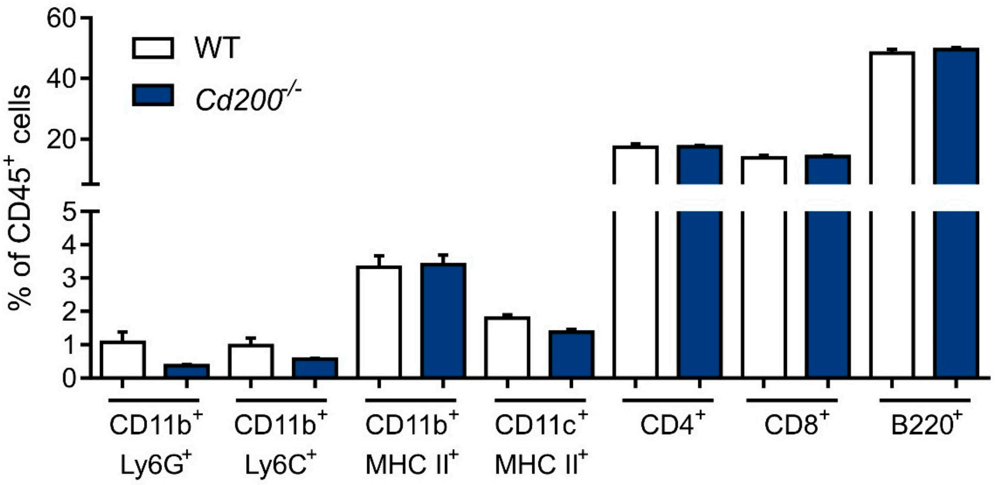

B

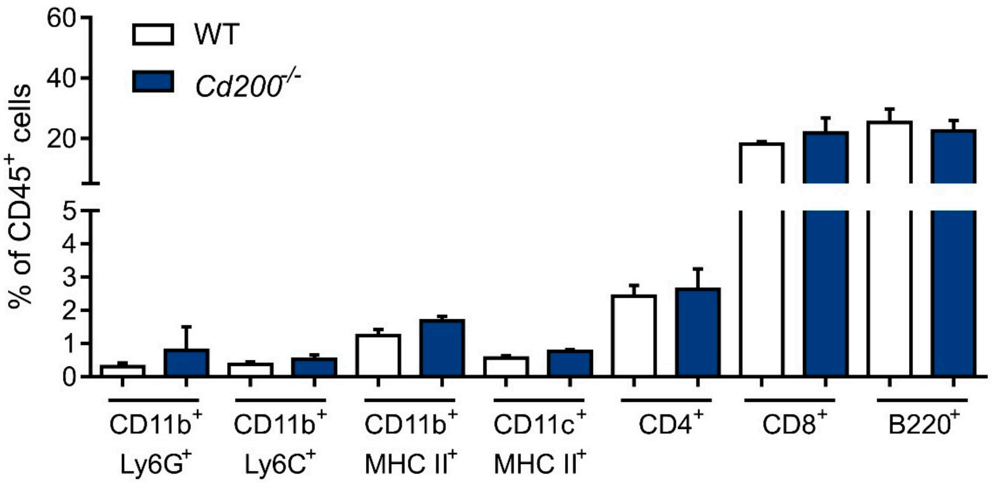

Figure S1. Lack of CD200 does not influence the number of myeloid cells and lymphocytes in the resting colon **(A)** and spleen **(B)** of conventionally reared mice.

Frequency of neutrophils ($CD11b^+Ly6G^+$) , monocytes ($CD11b^+Ly6C^+$), macrophages ($CD11b^+CD11c^-$ $MHC\text{-}II^+$), dendritic cells ($CD11b^-CD11c^+MHC\text{-}II^+$), $CD4^+$ T lymphocytes, $CD8^+$ T lymphocytes, and $B220^+$ B lymphocytes isolated from healthy colons or spleens of WT and *Cd200*$^{-/-}$ mice. Data represent mean proportions of cells within the live $CD45^+$ fraction, n=4.

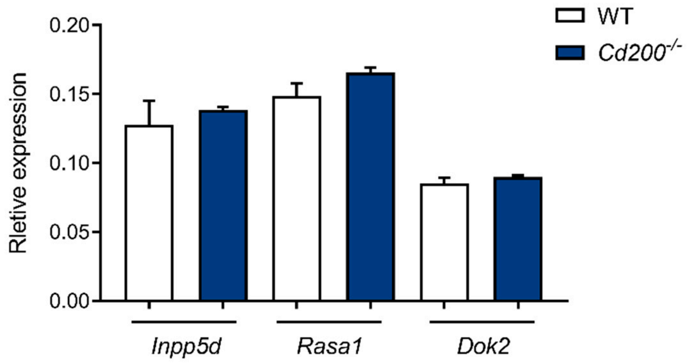

Figure S2. Lack of CD200 does not influence expression of *Inpp5d*, *Rasa1* or *Dok2*.

The expression of genes involved in CD200R singlaing including *Inpp5d* (encoding SHIP1), *Rasa1* (encoding p120RasGAP) and *Dok2* (encoding Dok2). RNA was isolated from colons of untreated WT and *Cd200*$^{-/-}$ mice, n=3. Genes were detected using specific TaqMan Gene Expression Assay from ThermoFicher Scientific according to the manufacrturers protocol. Each bar represents avegare (with SEM) from 3 independent biological replicates.

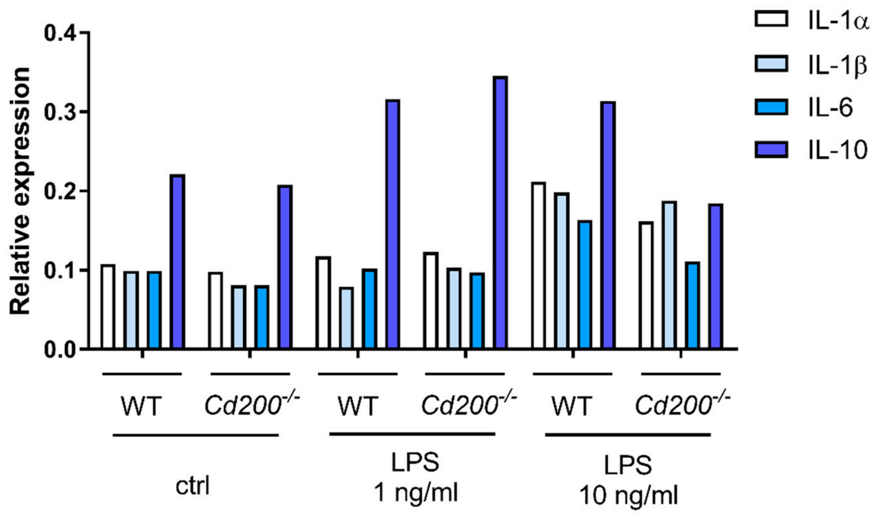

Figure S3. Lack of CD200 does not influence expression of IL-1α, IL-1β, IL-6, IL-10 in BMDMs in control contions nor upon LPS stimulation .

The expression cytokines produced by BMDMs (IL-1α, IL-1β, IL-6, IL-10). BMDMs were isolated from WT and *Cd200*$^{-/-}$ mice. Cells (n=2) were stimulated with 1 - 10 ng/ml LPS for 4h. Next cells were lysed, RNA was islated and used for detection of the cytokines. Genes were detected using specific TaqMan Gene Expression Assay from ThermoFicher Scientific according to the manufacrturers protocol. Each bar represents avegare from 2 independent biological replicates.

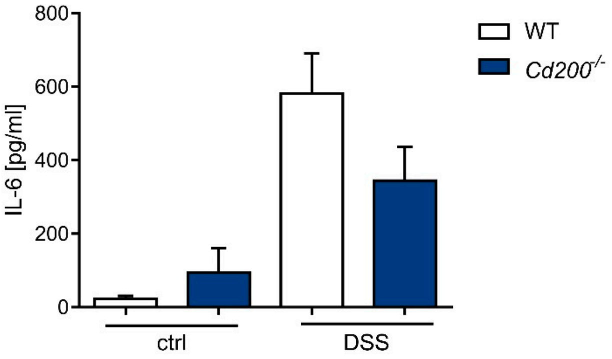

Figure S4. Lack of CD200 does not influence the amount of colonic IL-6.

The concentration of IL-6 in medium from 24h culture of colon explants isolated from control or DSS-treated WT and *Cd200*$^{-/-}$ mice, n=4-13. The concentration of IL-6 was measured in culture supernatants by ELISA (eBioscience or Invitrogen). ELISA was performed according to the manufacturer's protocol, with detection done using Asys UVM 340 Microplate Reader (Biochrom).
